# Mathematical models for thermionic emission current density of graphene emitter

**DOI:** 10.1038/s41598-021-01546-2

**Published:** 2021-11-18

**Authors:** Olukunle C. Olawole, Dilip K. De, Sunday O. Oyedepo, Fabian I. Ezema

**Affiliations:** 1 Department of Physics, Covenant University, Ota, Ogun State Nigeria; 2 Sustainable Green Power Technologies, San Antonio, TX USA; 3 Department of Mechanical Engineering, Covenant University, Ota, Ogun State Nigeria; 4 UNESCO-UNISA Africa Chair in Nanosciences-Nanotechnology, PO Box 392, Pretoria, South Africa; 5 Nanosciences African Network (NANOAFNET), iThemba LABS-National Research Foundation, PO Box 722, Somerset West, Western Cape Province South Africa; 6 Department of Physics and Astronomy, University of Nigeria, Nsukka, Nigeria; 7 Africa Centre of Excellence for Sustainable Power and Energy Development (ACE-SPED) University of Nigeria, Nsukka, Nigeria

**Keywords:** Devices for energy harvesting, Mathematics and computing, Nanoscience and technology, Optics and photonics, Physics, Energy science and technology, Energy harvesting, Renewable energy, Thermoelectric devices and materials

## Abstract

In this study, five mathematical models were fitted in the absence of space charge with experimental data to find a more appropriate model and predict the emission current density of the graphene-based thermionic energy converter accurately. Modified Richardson Dushman model (MRDE) shows that TEC's electron emission depends on temperature, Fermi energy, work function, and coefficient of thermal expansion. Lowest Least square value of $S=\sum {\left({J}_{th}-{J}_{exp}\right)}^{2}=0.0002 \,\text{A}^{2}/\text{m}^{4}$ makes MRDE most suitable in modelling the emission current density of the graphene-based TEC over the other four tested models. The developed MRDE can be adopted in predicting the current emission density of two-dimensional materials and also future graphene-based TEC response.

## Introduction

**Figure 1:**
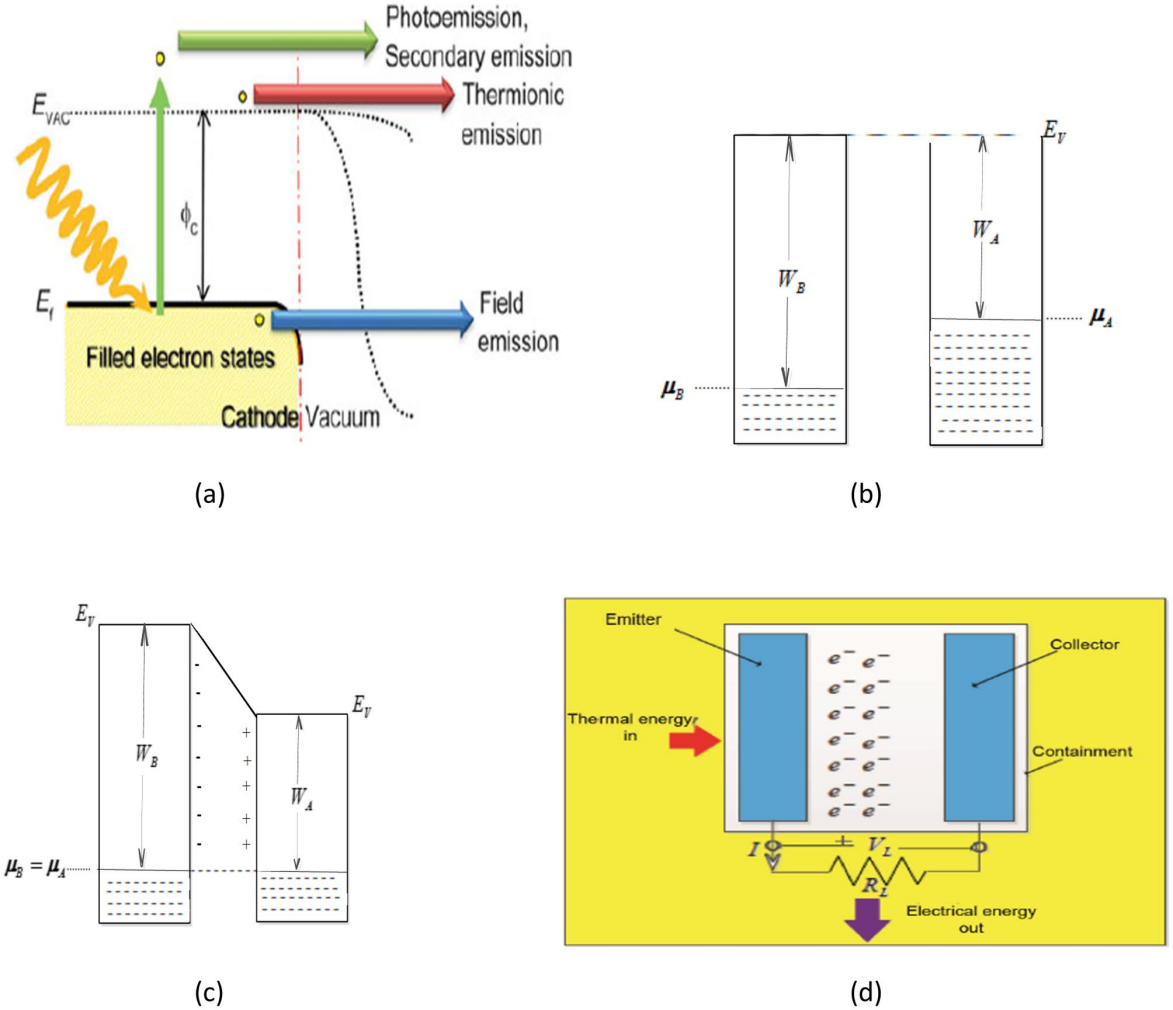
(**a**) Various mechanisms of producing electron emission^6,17,30^. (**b**) Unconnected cathode and anode^7^. Reprinted (**a**) with permission from Trucchi, D. M. & Melosh, N. A. Electron-emission materials: Advances, applications, and models. *MRS Bull.* (2017) https://doi.org/10.1557/mrs.2017.142. Copyright (2021) by the SpringerNature. (**c**) Connected cathode and anode^7^. (**d**) Thermionic energy converter set up^7^.

As a family member of two-dimensional (2D) materials, graphene remains an electrode of choice in the heart of researchers in harvesting electricity via thermionic and photo-enhanced thermionic energy converter due to its unmatched potentials^1–8^. Because of its unmatched prospects in electronics, thermal expansion, optical and mechanical properties, ease of tune-ability of its work function that is seen to operate in the 2D world^9–15^ and graphene's tolerance of high temperature^16^. Recently the mechanical properties were experimentally proven to use also in the 3D world^12,17^. In principle, Fig. 1a shows how the emitted electrons (working fluid) can be induced through thermal^6,7,18–20^, photo^4–6,21,22^, secondary^6,23^ and field emission^6,24–27^. The thermionically excited electrons work as the working fluid above the potential interface barrier and, after that, is collected at the anode of the TEC. Fundamentally, a TEC setup comprises a cathode (B), an anode (A), a conducting wire, and a load (Fig. 1d). And for this to perform optimally, the work function of electrode B must be greater than the work function of anode A; the temperature of B must be higher than the temperature of A. The distance between A and B must be of the order of micrometer to minimize the effects of space charge and thus, for the emitted electron to reach the collector and in the absence of space charge. However, when the electrodes in Fig. 1b are not electrically connected, its vacuum energy remains the same. Consequently, when electrodes in Fig. 1c are electrically connected, the alignment of chemical potential is noticed due to electrons migration from electrode A to electrode B. In addition, the presence of chemical potential in Fig. 1c creates a potential difference. Moreover, this potential difference in Fig. 1c stops further migration of electrons from anode to cathode. Nevertheless, as soon as the external load is attached to the setup in the presence of continuous supply of thermal energy from sunlight as depicted in Fig. 1d, the electrons at the cathode would have sufficient energy to overcome a surface barrier, migrate to the anode and drive electricity through the TEC.

Despite enormous research in TEC, the TEC technology's potential in generating electricity is still being hindered due to high material work function and space charge related problems^7,28–30^. This has led to the unnatural death of technology in the 90 s. Nevertheless, a recent revival of interests in thermionic energy converters is attributed to the emerging nanomaterials and ever-growing technology in the twenty-first century^5,6,8^. The technologies that are primarily of interest for applying graphene-based TEC are graphene's growth on solid tungsten and silicon carbide substrates^16,31–33^ and ease of work function engineering of graphene surface^13,15,34–36^.

Theoretically, researchers have seen green light about the terrestrial applications of TEC instead of space applications through the usage of nanomaterials as electrodes in TEC. In harnessing a graphene-based TEC's hidden electric power generation potential, specific parameters such as power output, thermal exchange, heat removal rate, and efficiency must be accurately modelled with some other TEC parameters.

### Power output of graphene-based TEC

**Figure 2:**
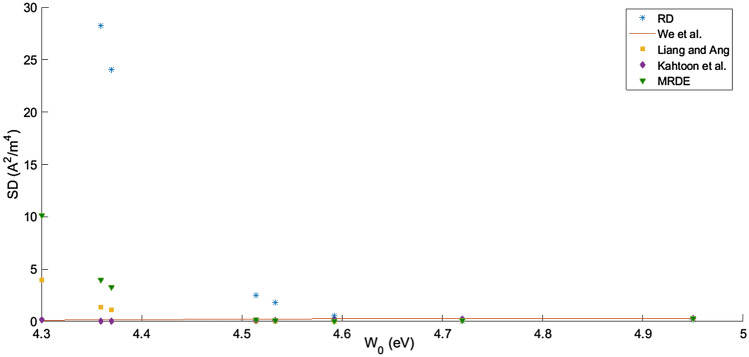
$SD (\text{A}^{2}/\text{m}^{4})$ against the work function (eV) of five models in TEC.

The formation of the potential barrier $\frac{\left({W}_{e} - {W}_{c}\right)}{e}$ (in the absence of space charge) in Fig. 1c is explained in detail in references ^7^ and ^8^. Once electrons are emitted thermionically at the emitter by overcoming the barrier due to its work function), the electrons find an electric field between the emitter and the anode due to the potential barrier $\frac{\left({W}_{e} - {W}_{c}\right)}{e}.$ These electrons would deliver power at the load ${P}_{0}$ as in Eqs. (1–2) (Fig. 2).

The present study aimed at examining the power output of graphene-based TEC specifically in the absence of space charge:

1 $${P}_{0}=\left({I}_{e}-{I}_{c}\right)\frac{\left({W}_{e} - {W}_{c}\right)}{e}$$

2 $${P}_{0}=\left({J}_{e}-{J}_{c}\right)s\left(\frac{{W}_{e} - {W}_{c}}{e}\right)$$

where ${I}_{e}$ is the thermionic current of the emitter, ${I}_{c}$ is the thermionic current collector, ${T}_{e}$ is the temperature of the emitter, ${T}_{c}$ is the temperature of the collector. In addition, ${W}_{e}$ is the of emitter's work function, ${W}_{c}$ denotes collector's work function, $e$ typifies electronic charge, and the power output is represented by ${P}_{0}$. However, ${J}_{e}$ is the emitter's current density, ${J}_{c}$ represents the collector's current density and *s* connotes the emitter's surface area.

### Fundamental equation for energy balance in TEC

So far, modelling a TEC has neglected heat radiation losses, affecting the efficiency calculation seriously, as shown by Olawole and De^7^. Incorrect modelling of efficiency of a TEC energy conservation which was not appropriately modelled except in Refs.^7,37^ must be taken fully into account. This is given for a solar TEC by Eq. (3)^38–40^.

3 $${I}_{0}\left(S-s\right)=\left[\left(\frac{{J}_{e}s\left({W}_{e}+2{k}_{B}{T}_{e}\right)}{e}-\frac{{J}_{c}s\left({W}_{c}+2{k}_{B}{T}_{c}\right)}{e}\right)+\left[\sigma s\left({T}_{e}^{4}-{T}_{a}^{4}\right)\right]+\left[\sigma s\left({T}_{e}^{4}-{T}_{c}^{4}\right)\right]\right]$$

where ${I}_{0}$ is the solar irradiance, S connotes the area of the parabolic concentrator, s is known as the emitter's area and $\sigma$ stands for Stefan Boltzmann's constant. Alternately, where the heat source is a burner or some other device, ${I}_{0}\left(S-s\right)$ is the incident total heat flux at the emitter.

Therefore, the first term on the right side of Eq. (3) ${J}_{e}s\left({W}_{e}+2{k}_{B}{T}_{e}\right)/e$ shows the total energy acquired by ejected electrons at the emitter. Likewise, the second term on the right hand of Eq. (3) $\left[{J}_{c}s\left({W}_{c}+2{k}_{B}{T}_{c}\right)/e\right]$ depicts the total energy electrons at collector absorbed. Furthermore, $\left[{J}_{e}\left(2{k}_{B}{T}_{e}\right)/e\right]$ denotes heat energy imparted on the collector by the emitted electrons from the cathode. Besides, $\left[{J}_{c}\left(2{k}_{B}{T}_{c}\right)/e\right]$ is the thermal energy the electrons emitted from an anode imparted on the cathode.

### Rate of thermal energy removal in TEC

In principle, the temperature of the collector plate rises fast due to the bombardment of the emitted electrons from the cathode. The thermal removal rate will be modelled with Eq. (4):

4 $${Q}_{r}=\left[\left(\frac{{J}_{e}\left(2{k}_{B}{T}_{e}\right)}{e}-\frac{{J}_{c}\left(2{k}_{B}{T}_{c}\right)}{e}+\sigma \left({T}_{e}^{4}-{T}_{c}^{4}\right)\right)s\right]$$

where ${Q}_{r}$ is the thermal removal rate from the collector to keep its temperature at T_c_ in the TEC system.

### The efficiency of TEC

The efficiency of graphene-based solar TEC can be modelled by Eq. (5):

5 $$\eta =\frac{\left({W}_{e}{ - W}_{c}\right)\left({J}_{e} -{J}_{c}\right)s}{{I}_{0}\left(S-s\right)e}$$

where ${I}_{0}\left(S-s\right)$ is the total solar power incident on the emitter. From Eqs. (3)–(5) can be deduced the influence of the parameters, ${I}_{o}$, S, s, T_e_ and T_c_, ${W}_{e},{W}_{c}$ on $\eta$. Thus, it is essential to correct J vs. T for nanomaterials to model a TEC. Equation (5) stands universal and applies to all TEC^7,8,37,41,42^. Graphene also has been found suitable for thermionic energy conversion^7,28,37,40,43–48^. Thus arises the importance of correct modelling of J vs. T for proper modelling of the efficiency of solar and other types of thermionic energy converters. For solar TEC Eq. (3) is found useful^7,37^. It is to be noted that for proper modelling of J vs T, the dependence of W on T must be taken into account. This aspect has been discussed earlier to some extent for TEC conversion of solar energy to generate electricity in future. More needs to be done, especially for nanomaterials.

The recent issue being raised in scientific communities is the fitness of the Richardson Dushman (RD) equation that was enacted for metals^18,28,49^ only in predicting and determining the current density of nano-thermionic energy^6,20,47^ converter. In response to the fitness of the RD equation, some scholars have affirmed the potency of the RD equation in determining the current density of the emitters and collectors in nano-thermionic engines^58–60^. At the same time, some researchers opined that using macroscopic RD law to investigate a nano-thermionic energy converter (NTEC) is against the physics of nanoscience and nano-engineering^7,20,37,50–52,61^. To resolve this challenge, the experimental results of Zhu et al.^44^ for J vs. T in graphene have been adopted to fit the various models and to find the most suitable model for predicting the accuracy of the current density of graphene-based thermionic energy converter.

## Methodology

This study has acquired graphene's experimental thermionic emission (J vs. T) data from reliable and standard source^44^. Consequently, the five theoretical models (Eqs. 36–40) were coded in MATLAB to fit the experimental current density of graphene-based TEC.

### Richardson–Dushman model

Nobel Prize-winning Eq. (6) of Richardson-Dushman was explained on the theory of Sommerfeld for probing the mechanism of electron emission in electronic devices. Equation (6) opined that the current density of the thermionic converter is a subject of the work function and temperature of the metal^49^:

6 $$J = A_{0} T^{2} \exp \left( { - \frac{W}{{k_{B} T}}} \right)$$

Typically, ${A}_{0}=\frac{{4\pi emk_{B}^{2} }}{{h^{3} }}$ denotes Richardson-Dushman value $1.2\times {10}^{6} \,\text{Am}^{-2}\,\text{K}^{-2}$, $W$ stands for work a function, T is the temperature, e is the electronic charge, m is the electron mass, h typifies the Planck's constant, and ${k}_{B}$ connotes the Boltzmann constant.

### Wei et al. and Kim and Lee model

Investigation of Wei et al.^52^ confirmed that Sommerfeld theory could have been a suitable model in predicting electron emission from the edge of two-dimensional (2D) materials like graphene. However, the current density from the edge is too small to be of any practical importance. Hence, the scientific thought of Wei et al.^52^ and Kim and Lee^53^ on the electron emission from the graphene sheet with atomic thickness is expressed as:

7 $$J = A_{0} T^{3/2} \exp \left( { - \frac{W}{{k_{B} T}}} \right)$$

where ${A}_{0}=\left[{\left(m/2\right)}^{1/2}{\left({k}_{B}/\pi \right)}^{3/2}\left(e\right)/{\left(\hslash \right)}^{2}\right]= 2.2573\times {10}^{4}$ $A{\mathrm{m}}^{-2}\,{\mathrm{K}}^{-3/2}$ is the Richardson-Dushman constant for graphene, W is the work function of the material, T is the temperature, and ${k}_{B}$ is the Boltzmann constant.

Moreover, a complexity in their current density versus temperature model when tunnelling effect is applied at a different energy level and time interval in examining the confinement of electrons in a potential barrier poses a fundamental question on the correctness of their theory. Also, this theory should be conceived in the heart and not in the practical term.

### Liang and Ang model

The theoretical investigation of Liang et al.^20^ showed that the RD is a non-valid theory to examine the current emission density of graphene. Their study considered the in-plane mass of graphene electrons as a zero entity. Even though they thought the finite mass of electrons for motion perpendicular to the graphene surface, they got rid of this finite mass in the final equation using some questionable tricks. They assumed a temperature-independent work function. This allows their study to adopt a new Richardson Dushman constant of $115.8 {\mathrm{Am}}^{-2}\,{\mathrm{K}}^{-3}$. On the contrary, Yoon et al.^54^ experiment revealed the non-massless nature of graphene when the estimated dynamical electron mass of graphene is found between 0.01*m*_*e*_ to 0.024*m*_*e*_, which raises doubt on Liang and Ang idea^20^ in Eq. (8).

They did not consider the discrete nature of electron energy perpendicular to the graphene surface. In their theory, they put the lower limit of integration for the energy of emitted electron as $\phi$ whereas it should be ${E}_{F}+\phi$, which is the minimum energy of the electron which can be emitted. The energy of the electron at that point is ${E}_{F}+\phi$ and not just $\phi .$

Consequently, their findings opined that the current density of graphene-based TEC is dependent on a cube of its temperature and work function and Fermi energy, as shown in Eq. (38)^20^

8 $$J = \frac{{ek_{B}^{3}T^{3} }}{{\pi \hbar^{3}v_{F}^{2} }}\exp \left( { - \frac{{W - E_{F} }}{{k_{B} T}}} \right)$$

${A}_{0}=\frac{{ek_{B}^{3}}}{{\pi \hbar^{3}v_{F}^{2} }}$ is the assumed Richardson Dushman constant for graphene. $=115.8  A{m}^{-2}{K}^{-3}$. Where W is the work function of the material, ${E}_{F}$ is the Fermi energy ${v}_{F}$ is the Fermi velocity, T is the temperature, e is the electronic charge, $\hslash$ is the reduced Planck's constant, and ${k}_{B}$ is the Boltzmann constant. In their Eq. (8), they have treated ${E}_{F}$ and ${v}_{F}$ as two separate items contradictory to Eq. (9):

9 $${E}_{F}= {m}_{c}{V}_{F}^{2}$$

### Khatoon Ansari and Ashraf model

Consequently, Khatoon et al. considered how to work function and Fermi energy are a function of temperature in^50^:

10 $$J = \frac{{ek_{B}^{3}T^{3}}}{{\pi\hbar^{3}v_{F}^{2} }}\exp \left( { - \frac{{\left[ {\left( {\phi \left( 0 \right) + \mu \left( 0 \right)\left( {1 - \alpha T} \right)} \right) - \left( {{{\mu \left( 0 \right)} \mathord{\left/ {\vphantom {{\mu \left( 0 \right)} {2\ln 2}}} \right. \kern-\nulldelimiterspace} {2\ln 2}}} \right)\left( {{{T_{F} } \mathord{\left/ {\vphantom {{T_{F} } T}} \right. \kern-\nulldelimiterspace} T}} \right)} \right]}}{{k_{B} T}}} \right)$$

${A}_{0}=\frac{{ek_{B}^{3}}}{{\pi \hbar^{3}v_{F}^{2} }}$ is an assumed Richardson Dushman constant for graphene and equals $115.8  A{m}^{-2}{K}^{-3}$ as in Liang and Ang model. Where $\phi (0)$ is the work function of the material at absolute zero temperature, $\alpha$ is the coefficient of thermal expansion, $\mu (0)$ is the Fermi energy at absolute zero temperature, ${v}_{F}$ is the Fermi velocity, T is the temperature, ${T}_{F}$ is the Fermi temperature, e is the electronic charge, $\hslash$ is the reduced Planck's constant, and ${k}_{B}$ is the Boltzmann constant.

### De and Olawole model

De and Olawole remarked that three dimensional (3D) approach has the potential to model the mechanism of thermionic emission in 2D electrodes(graphene) based on the following conditions^37^:

- That Fermi energy ${E}_{F}(T)$, work function (W) and thermal expansion of 2D electrodes are a function of temperature.
- Thermionic emission must be perpendicular to the graphene surface. For in-plane motion, the electrons must possess in-plane momentum components ($${k}_{x}$$, $${k}_{y}).$$ For emission normal to the z-plane, the electron must possess $${k}_{z}$$ component of momentum. Thus, the electron must possess three components of momentum for thermionic emission from the 2-D graphene.
- Re-filling of the vacant energy sites created by the emitted electrons at emitter with electrons, through back electrons from the anode to cathode.
- z-component of graphene-based thermionic emission possesses momentum in the 3D world provided $W+{E}_{F}\le \left|\frac{{P}_{z}^{2}}{2m}\right|\le \infty$.
- It has also been noted that the generation of electricity through edge emission is impracticable^52^ as the current will be too small. In addition, the following scenarios that dictate the emissions of electrons from graphene along z-direction need to be considered:
- Electrons in z-plane possess discrete energy levels as particles which may correspond either to an infinitely square well potential or a finite square well such that though the electron motion in the plane of graphene is massless corresponding to the electron dispersion relation: $E(q) = \pm {V}_{F}\left|q\right|$; however, it possesses finite mass for motion along the z-direction. Electron emission takes place when it contains energy more significant than the work function of the material.
- Let's consider electron energy levels to be dictated by the finite square well. Then, tunnelling probability must be regarded as allowing emission to occur, especially when the energy of tunnelling electrons is higher than the work function of the confinement.

So far, no model has considered all the above realistic scenarios of dynamics of electron emission from the 2-D graphene, as it is too complicated to handle theoretically.

To avoid the complication associated with a near-perfect model as mentioned above, considering no. (v) De and Olawole assumed a 3D model (due to three momentum of the electrons) considering thermal expansion of the lattice and temperature dependence of work function. The 3D model of graphene is recently found to hold for mechanical properties of graphene^12^. They also considered the temperature dependence of work function. With these considerations, the Richardson Dushman equation was modified to model the current density of graphene in terms of Fermi energy and work function dependent on temperature^7,8,37,45^.

11 $$J = A_{0} T^{2} \exp \left( {{{ - \left[ {W_{0} + \left( \begin{gathered} [r\alpha T + \left( {1 + r\alpha T} \right)\left( {\frac{{\pi^{2} }}{12}} \right)\left( {\frac{{k_{B} T}}{{E_{F0} }}} \right)^{2} ]E_{F0}  + \left( {1 + r\alpha T} \right)\left( {\frac{{7\pi^{4} }}{960}} \right)\left( {\frac{{k_{B} T}}{{E_{F0} }}} \right)^{4} \left( {E_{F0} } \right) \end{gathered} \right)} \right]} \mathord{\left/ {\vphantom {{ - \left[ {W_{0} + \left( \begin{gathered} [r\alpha T + \left( {1 + r\alpha T} \right)\left( {\frac{{\pi^{2} }}{12}} \right)\left( {\frac{{k_{B} T}}{{E_{F0} }}} \right)^{2} ]E_{F0}  + \left( {1 + r\alpha T} \right)\left( {\frac{{7\pi^{4} }}{960}} \right)\left( {\frac{{k_{B} T}}{{E_{F0} }}} \right)^{4} \left( {E_{F0} } \right)  \end{gathered} \right)} \right]} {k_{B} T}}} \right. \kern-\nulldelimiterspace} {k_{B} T}}} \right)$$

${A}_{0}=\frac{{4\pi emk_{B}^{2} }}{{h^{3} }}$ is the Richardson-Dushman constant for graphene = ${1.2\times 10}^{6} \,\text{Am}^{-2}\,\text{K}^{-2}$. Where W_0_ is the work function of the material, r is the dimensionality of the material, such that 2D graphene, has a r value of 2 while carbon nanotube has a r value of 1, T is the temperature, $\alpha$ is the coefficient of thermal expansion, e is the electronic charge, m is the electron mass, h typifies the Planck's constant, ${E}_{F0}$ is the Fermi energy, and ${k}_{B}$ is the Boltzmann constant.

## Results and discussion

Table 1 depicts how different parameters $\left({W}_{0,} {E}_{F0,}{V}_{F}\right)$ affect the various least-square values for the five models. The least-square method is used to select work function, Fermi energy, and Fermi velocity that fits most of the thermionic emission current density data (experimental) of 2D materials (graphene) with the model's theoretical values the model could be the best.

**Table 1:** Influence of work function, Fermi energy, and Fermi velocity on the current density of graphene as dictated by the square difference (SD).

Models	Year	${\mathrm{W}}_{0} (\mathrm{eV})$	${\mathrm{E}}_{\mathrm{F}0}$ $(\mathrm{eV})$	${\mathrm{V}}_{\mathrm{F}}(m/s)$ ${10}^{6}$	SD = $({\mathrm{A}}^{2}/{\mathrm{m}}^{4})$
Richardson Dushman	1928	4.720	–	–	0.0006
Wei et al	2013	4.300			0.1170
Liang and Ang	2015	4.533	0.083	$1.000$	0.0004
De and Olawole	2018	4.592	0.203	–	0.0002
Kahtoon et al	2018	4.370	0.083	1.000	0.0053
		4.514	0.083	2.49	0.0984

**Figure 3:**
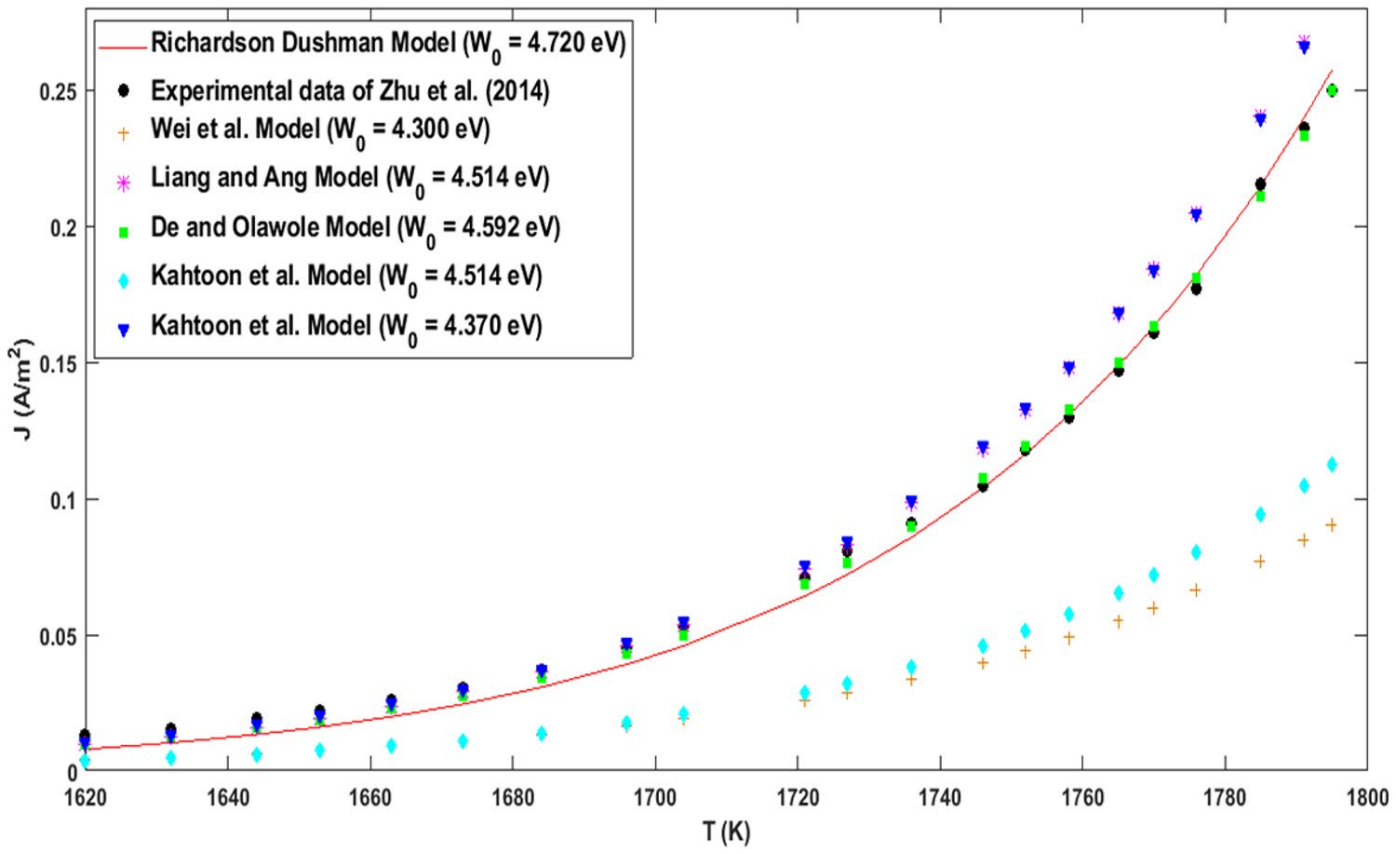
Best fitting of the theoretical thermionic emission current density of graphene with experimental data (black dotted points) for different models of graphene. The solid red line for Eq. (6) (RD model), yellow cross for Eq. (7) (Wei et al., model), magneta solid for Eq. (8) (Liang and Ang model), cyan diamond and blue triangular for Eq. (10) (Khatoon et al., model), and green square for Eq. (11) (De and Olawole model).

Figure 3 shows the best fit of the experimental data of J vs. T with different models. Interestingly, the solid red line of Eq. (6) slightly fits the experimental black dotted points at ${W}_{0}=4.720 \,\text{eV}$ with a least-square value of $0.0006 \text{A}^{2}/\text{m}^{4}$. In addition, the yellow cross of Eq. (7) in Fig. 3 initially deviates slightly from the experimental black dotted points at 1620–1755 K and later fits at 1760–1795 K. Its least-square value is $0.1170 \,\text{A}^{2}/\text{m}^{4}$ with tuned ${W}_{0}=4.300 \,\text{eV}$. Consequently, the magenta star of Eq. (8) in Fig. 3 fits the experimental black dotted points at 1620–1730 K and deviates fairly at 1740–1795 K. Its least-square value is $0.0004\,\text{A}^{2}/\text{m}^{4}$ with tuned ${W}_{0}=4.533\, \text{eV}$, ${E}_{F0}=0.083\, \text{eV}$ and ${V}_{F}=1\times {10}^{6} \text{m/s}$. The cyan diamond of Eq. (9) in Fig. 3 deviates slightly from the experimental black dotted points from $1620 \,\text{K}$ up to 1795 K with a least-square value of $0.0053 \,\text{A}^{2}/\text{m}^{4}$, ${W}_{0}=4.3700\, \text{eV}$, ${E}_{F0}=0.083 \,\text{eV}$ and ${V}_{F}=1\times {10}^{6} \,\text{m/s}$.

While the green square of Eq. (11) in Fig. 3 shows a good fit with the experimental black dotted points at ${W}_{0}=4.592 \,\text{eV}$, ${E}_{F0}=0.203 \,\text{eV}$ which results in the least-square value of $0.0002\,\text{A}^{2}/\text{m}^{4}$. Statistically and graphically, the representation of Table 1 and Figs. 2 and 3 have shown that Eq. (11) modelled experimental data^44^ of graphene accurately as its electron emission depends on temperature, Fermi energy, work function, and coefficient of expansion. That graphene's Fermi energy is temperature-dependent, precisely at 0 K, which was ignored in Eqs. (6–8, 10)^20^.

We propose the fitting of De and Olawole model^37^ (Eq. 11) to thermionic emission data from graphene based structures. Recent studies^55–57^ on graphene based Schottky barrier junction (lightly doped graphene with metal junction) show prospect of applying the equation in which the external voltage induced diode current density $J$ is governed by the saturation current density $({J}_{0})$. Also, ${J}_{0}$ is thought to be of thermionic origin and according to various authors different forms are given according to Javadi et al.^57^ in Eq. (12).

12 $${J}_{0}={C}_{G}\left(q{\phi }_{B}\right){v}_{\perp }^{*}Texp\left(-\frac{q{\phi }_{B}}{{k}_{B}T}\right)$$

where ${C}_{G}$ is a constant related to thermionic property of graphene and is shown to be $0.06 C/eV{cm}^{3}K$^57^; ${\phi }_{B}$ is the Schottky barrier height, and ${v}_{\perp }$ is the out of plane electron velocity.

Consequently Sinha and Lee^56^, stated saturation current density $\left({J}_{0}\right)$ as:

13 $${J}_{0}={A}^{*}{T}^{2}exp\left(-\frac{q{\phi }_{B}}{{k}_{B}T}\right)$$

Kalita et al.^55^ gave the relation for saturation current density in graphene-silicon Schottky barrier diode same as in Eq. (13). However, none of these authors gave the experimental ${J}_{0}$ against T data and for the $J$ against $V$ graphs temperature $\left(T\right)$ was not mentioned by Kalita et al.^55^ and Sinha and Lee^56^. In addition, Javedi et al.^57^ however mentioned temperature for their $J$ against $V$ (external voltage) curves but it is hard to extract the ${J}_{0}$ against T data which ideally should be external voltage independent. Thus, more experiments on such diodes are needed to extract ${J}_{0}$ against T data on which our model (MRDE) can be conveniently tested, whether it supports or fails. It would be an interesting field of research.

## Conclusion

This study has discussed various models, including the present work results, and compared them in fitting the experimental results of thermionic emission current density versus temperature data for 2D graphene. We have given detailed reasons for the problems associated with a perfect physics model that should explain the massless nature of thermionic electron for motion in the 2D plane of graphene, finite mass, and discrete quantized energy levels for motion along the $z$-direction (thermionic emission). The study presents how various models failed to take care of these essential facts and the generation of momentum components along $z$-direction of thermionic electrons. In this paper, we have given a brief explanation of why a 3D dimensional model for thermionic emission from a 2D graphene may be appropriate, especially in the absence of a perfect physics model in literature, and we have considered very detailed comparisons of the previous models with our model that was not reported by us earlier.

This study also reveals how the Richardson Dushman model^49^ fails to harmonize the experimental and theoretical results of current density vs T with published works. Also, Wei et al. model^52^, which was discussed qualitatively, is too difficult to handle. It would require electrons tunnelling through barriers of different widths and with varying times of relaxation (for tunnelling probabilities). Moreover, it is not easy to model the tunnelling times because of variation with quantized energies^51^. Thus, we observe a real challenge in formulating the current density-temperature relation of 2D graphene, considering the energy dispersion in the 2D plane and the discrete energy levels in the direction normal to the 2D plane of graphene.
